# Felix Battistella, UC Davis Chief of Trauma and Emergency Surgery, Editorial Board member of *World Journal of Emergency Surgery*, dies at 48, January 22 2008

**DOI:** 10.1186/1749-7922-3-12

**Published:** 2008-03-07

**Authors:** Cheryl Wraa RN, Fausto Catena, Ernest Moore

**Affiliations:** 1 MSN Trauma Program Manager University of California, Davis Medical Center, 2315 Stockton Blvd, Sacramento, CA 95817, USA; 2 Editor-in-Chief – *World Journal of Emergency Surgery*, St Orsola – Malpighi University Hospital, Bologna, Italy; 3 Editor-in-Chief – *World Journal of Emergency Surgery*, Chief of Surgery Denver Health/University of Colorado, Colorado, USA

Felix D. Battistella, Professor and Chief of Trauma and Emergency Surgery at UC Davis Medical Center, died from cancer on January 22 at his home. He was 48.

"*Dr. Battistella's unselfish dedication to his field, his colleagues and to the UC Davis Health System community was widely known*." said Claire Pomeroy, Vice Chancellor for Human Health Sciences and Dean of the UC Davis School of Medicine. "*The many contributions he made have forever shaped the way we provide compassionate care for patients in their hour of need*."

**Figure 1:**
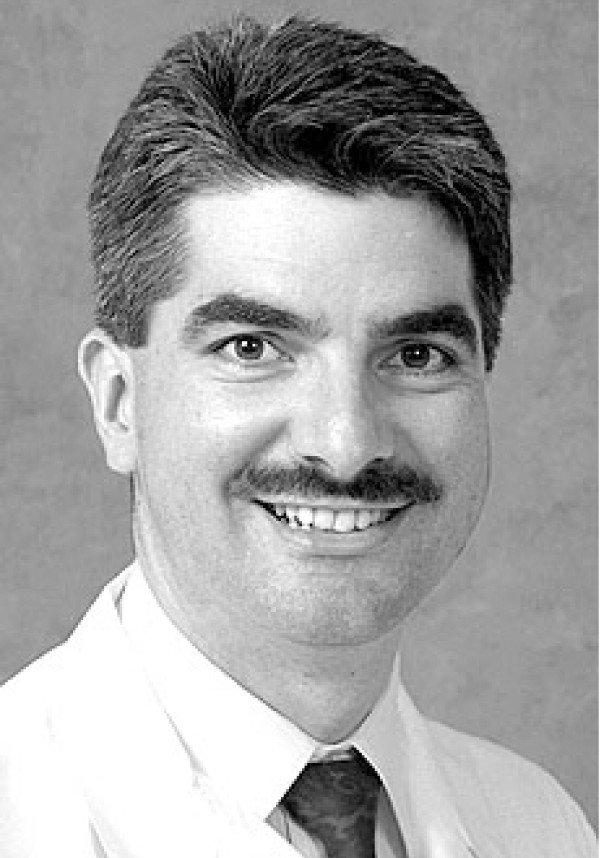
A photograph of the late Felix D. Battistella.

Battistella (figure 1) was born on September 13, 1959, in Monterey, California. After graduating from Monterey High School in 1977, he received a bachelor's degree in chemistry from the University of Santa Clara, where he graduated *magna cum laude*. He received his medical degree from the UC Davis School of Medicine in 1985. For his postgraduate training, Battistella continued his association with UC Davis, completing an internship in the Department of Surgery in 1986, and finishing his residency in the department in 1990. He served as Chief Resident in the department from 1990 to 1991.

Battistella joined the Faculty of the Department of Surgery as an Assistant Professor in 1991. In addition to serving as Professor and Chief of Trauma and Emergency Surgery, Battistella was the Director of the department's residency program. He also served as the Chief of Staff from 2004 to 2006.

In 1991, the senior class at the School of Medicine selected Battistella for its Outstanding Resident Teaching Award. That same year, he was voted Outstanding Chief Resident by his fellow residents in the Department of Surgery. In 1997, the Friends of Nursing at UC Davis Medical Center chose Battistella as its Physician of the Year.

Carol Robinson, Senior Associate Director, Patient Care Services Administration, said, "*Dr. Battistella will be remembered most by any nurse who worked with him as the kindest, most compassionate surgeon. He was a highly skilled surgeon, but what set him apart was that he treated every one of his patients with compassion and caring. All of our nursing staff greatly admired Dr. Battistella*."

Cheryl Wraa, Manager of the UC Davis Trauma Program, said, "*Dr. Battistella was a great teacher. In working with him and watching him with residents and students, you could see that he had a passion for teaching and mentoring. He also truly cared about the well-being of his patients*."

Battistella is survived by his wife, Christine; daughters Claire and Mary; his mother, Amalia; and his sister, Nedra.

We think that *World Journal of Emergency Surgery *lost a great Editor and all emergency surgery science lost a great contributor.

